# Multiple Modes of Impulsivity in Parkinson's Disease

**DOI:** 10.1371/journal.pone.0085747

**Published:** 2014-01-21

**Authors:** Cristina Nombela, Timothy Rittman, Trevor W. Robbins, James B. Rowe

**Affiliations:** 1 Department of Clinical Neurosciences, Cambridge University, Cambridge, United Kingdom; 2 Department of Psychology, Cambridge University, Cambridge, United Kingdom; 3 Behavioural and Clinical Neuroscience Institute, Cambridge, United Kingdom; 4 Medical Research Council, Cognition and Brain Sciences Unit, Cambridge, United Kingdom; Hospital General Dr. Manuel Gea González, Mexico

## Abstract

Cognitive problems are a major factor determining quality of life of patients with Parkinson's disease. These include deficits in inhibitory control, ranging from subclinical alterations in decision-making to severe impulse control disorders. Based on preclinical studies, we proposed that Parkinson's disease does not cause a unified disorder of inhibitory control, but rather a set of impulsivity factors with distinct psychological profiles, anatomy and pharmacology. We assessed a broad set of measures of the cognitive, behavioural and temperamental/trait aspects of impulsivity. Sixty adults, including 30 idiopathic Parkinson's disease patients (Hoehn and Yahr stage I–III) and 30 healthy controls, completed a neuropsychological battery, objective behavioural measures and self-report questionnaires. Univariate analyses of variance confirmed group differences in nine out of eleven metrics. We then used factor analysis (principal components method) to identify the structure of impulsivity in Parkinson's disease. Four principal factors were identified, consistent with four different mechanisms of impulsivity, explaining 60% of variance. The factors were related to (1) tests of response conflict, interference and self assessment of impulsive behaviours on the Barrett Impulsivity Scale, (2) tests of motor inhibitory control, and the self-report behavioural approach system, (3) time estimation and delay aversion, and (4) reflection in hypothetical scenarios including temporal discounting. The different test profiles of these four factors were consistent with human and comparative studies of the pharmacology and functional anatomy of impulsivity. Relationships between each factor and clinical and demographic features were examined by regression against factor loadings. Levodopa dose equivalent was associated only with factors (2) and (3). The results confirm that impulsivity is common in Parkinson's disease, even in the absence of impulse control disorders, and that it is not a unitary phenomenon. A better understanding of the structure of impulsivity in Parkinson's disease will support more evidence-based and effective strategies to treat impulsivity.

## Introduction

Impulsivity is common in many developmental, psychiatric and neurological disorders, including Parkinson's disease. Despite the prominence of impulsivity, it remains an heuristic construct, encompassing a wide range of acts that reflect poor cognitive control. Impulsive acts are poorly conceived or without foresight, prematurely executed, unduly risky or inappropriate to the situation, often with undesirable consequences [1].

Impulse control disorders affect up to one in seven people with Parkinson's disease and are potentially worsened by common dopaminergic therapies [2], [3]. Their importance has been emphasised by the operationalization of impulse control disorders in DSM-IV and the development of screening criteria for impulse control disorders in Parkinson's disease. Such clinical diagnoses complement a translational cognitive neuroscience approach to impulsivity, identifying the mechanisms of behavioural control and inhibition of actions, the regulation of behavioural strategies and processing of risk or reward [4]–[8]. For example, impulsivity may be tested in terms of refraining from, or cancelling, on-going movements (NoGo and Stop-Signal tasks respectively); looking away from a stimulus (anti-saccade); adhering to a less potent cognitive set (Stroop task); waiting for larger long term rewards, eschewing short term smaller gains (inter-temporal choice/temporal discounting); or moderating behaviours appropriately when outcomes are uncertain (gambling tasks). Neuropsychological studies of lesions and neuroimaging studies have identified critical anatomical substrates for such tests of impulse control [4], [9]–[11], emphasising especially the inferior frontal gyri, medial frontal cortex and anterior cingulate cortex, as well as regions of the striatum.

There is also evidence of psychopharmacological dissociations among different forms of impulsivity (see summary table 1). For example, the restraint (NoGo) has been associated with serotonergic function in human and comparative studies [12], [13], whereas cancellation (Stop signal task) has been associated with noradrenergic function [1], [5], [14]. Dopamine has been strongly associated with reward processing [2], [15], [16] and clinical impulse control disorders in Parkinson's disease [17]. These neurochemical dissociations are retained in the context of developmental and psychiatric disease [11], [18]–[21]. Indeed, impulsivity can be worsened by dopaminergic therapies [22]–[24] or deep brain stimulation [25], even in bradykinetic patients [26].

**Table 1:** Summary of neurotransmitter associations with performance on tests of inhibition or impulsivity.

Test		DA		NA		5HT		PFC
BIS	**++**	Halbig et al. [98]	−	Brambilla et al. [99] ∞	−	Marazziti et al. [100] h	**++**	Halbig et al. [98]
Stroop	**++**	Djamshidian et al. [101]	−	Spencer et al. [102]	**+** −	Boggio et al. [103]	**++**	Fera et al. [104]
Kirby	**++**	Housden et al. [105]	−	Mobini et al. [106] *	**++**	Nakahara et al. [107] h	**++**	Peters and Buchel [108] h
Go/NoGo	**++**	Antonelli et al. [109]	−−	Eagle et al. [14] h *	**++**	Schirmbeck et al. [110]	**++**	O'Callaghan et al. [111] ∞
Hayling	**++**	Lord et al. [112]	#		**+**	Raust et al. [113] ∞	**++**	Nathaniel-James et al. [114] h
SSRT	−	Bari and Robbins [115] *	**++**	Bari et al. [116] *	**+**−	Overtoom et al. [117] ∞	**++**	Eagle et al. [14]
MIDI	**++**	Weintraub et al. [22]	#		**++**	Lee et al. [64]	**++**	Isaias et al. [118]
BIS-BAS	−	Antonini et al. [119]	#		**+**	Cools et al. [120]	**++**	Cherbuin et al. [121] h
Gambling	**++**	Cools et al. [122]	**+**	Rotondo et al. [123]	**+**	Cools et al. [122] ∞	**++**	Cilia et al. [124]
Time Estimation	**++**	Perbal et al. [78]	#		**+**	Buhusi and Meck [125] *	**+**	Jones et al. [126]
South Oak Scale	**+**−	Isaias et al. [118]	#		**++**	Zapata et al. [127] *	**+**	Marazziti et al. [128] h
Saccadometre	**+ −**	Temel et al. [129]	#		#		++	Barker and Michell [93]

+: Evidence of significant influence of the neurotransmitter on the test score.

−: Evidence of probable lack of influence of the neurotransmitter on the test score.

+−: Inconsistent or equivocal evidence of influence of the neurotransmitter on the test score.

^#^ Lack of evidence of any specific effect of the neurotransmitter on the test score (for details of each test, see Table 2).

Citations correspond to studies in PD patients, otherwise marked as,

Patients with other diseases;

^h^ healthy controls;

rodents.

The question therefore arises, what are the components of impulsivity in Parkinson's disease? Many studies have examined selective aspects of impulsivity in Parkinson's disease, and a “generalized inhibitory deficit in Parkinson's disease” has been proposed [27]. This might reflect impairment of a single core mechanism for impulse control. Alternatively, Parkinson's disease may cause a multifaceted impairment of inhibitory control, resulting in a frequent but multidimensional disorder of impulsivity. From previous work it is clear that Parkinson's disease impairs restraint and cancellation of responses [28]; increases risky gambling [29], [30]; increases impulsive decision making ‘on’ medication [31]; impairs anti-saccades [32]; and distorts the perception of time leading to impatience [33]. Group level studies of Parkinson's disease also demonstrate significant changes in the associated neurotransmitter systems (including dopamine [34]–[36], noradrenaline [37], serotonin [38]–[40]) and anatomical networks in the frontal lobe for impulse control [41], [42].

**Figure 1:**
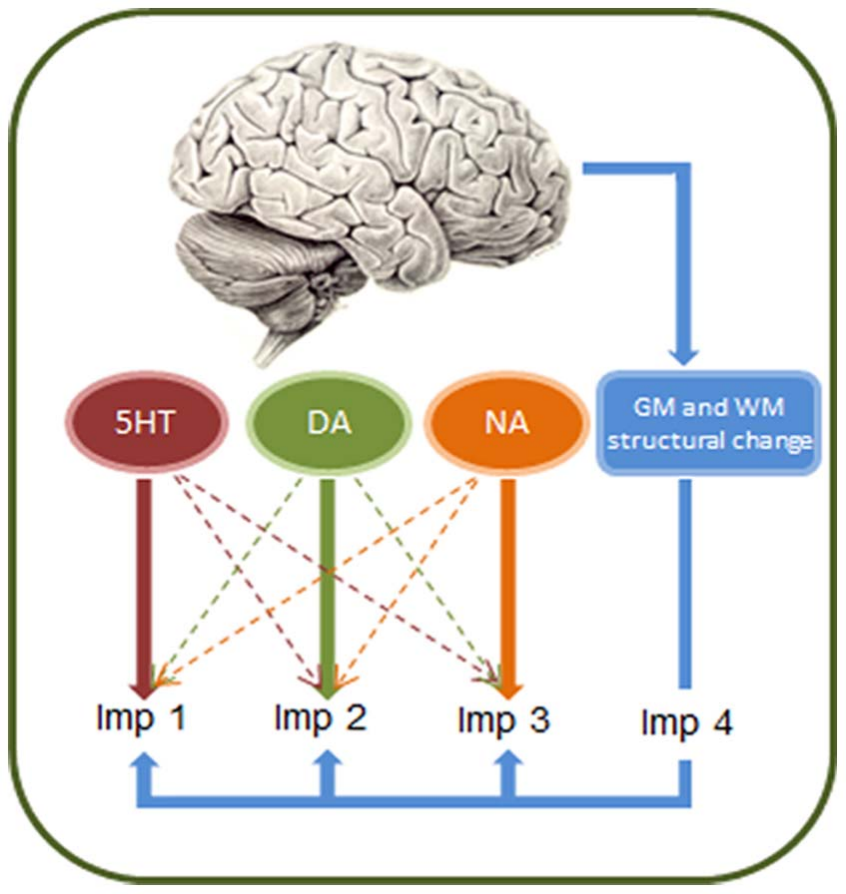
The multiple modes of impulsivity model. We propose that there are multiple modes of impulsivity in Parkinson's disease (Imp1, Imp2, etc.) which are driven by differential changes in brain structure (white matter, WM, and grey matter, GM), and changes in noradrenergic (NA), cholinergic (Ach) and dopaminergic (DA) neurotransmission. These separate modes reflect the ontology of impulsivity in health, and distinctive neural circuits for impulse control with partially selective pharmacology.

Our hypothesis was that the variances of dopamine [35], noradrenaline [37], serotonin [40] and atrophy could lead to separate ‘impulsivities’ in the context of Parkinson's disease (Figure 1). We therefore used an extensive battery of complementary tests of impulsivity/inhibition, many of which have been used to study Parkinson's disease, but not previously in the same group of participants. We then applied a factor analysis to the main outcome measures from these tests. This method assumes that covariation in observed variables is due to the presence of one or more underlying ‘latent variables’. We predicted the presence of at least four factors, reflecting the distinct anatomical and neurochemical systems for impulse control.

## Methods

Sixty adults were recruited and provided written informed consent. Their demographic and clinical features are summarized in Table 2. The sample included thirty patients with idiopathic Parkinson's disease from the John van Geest Centre for Brain Repair (University of Cambridge) Parkinson's disease research clinic. Patients met the United Kingdom Parkinson's Disease Society Brain Bank diagnostic criteria for Parkinson's disease [43]. Additional inclusion criteria were: no dementia at last clinical assessment and current Mini-Mental State Examination score ≥26/30 [44], and no current major depression [clinical impression and Beck Depression Inventory score ≤18, BDI-II [45]], to reduce the potential confounding effects of significant depression. Each patient's current drug regime was converted to an equivalent levodopa daily dose to facilitate further comparison [46]. All testing was performed with patients taking their usual medication. Thirty age-matched controls were recruited from the Medical Research Council Cognition and Brain Sciences Unit volunteers' panel. The Cambridge Local Research Ethics Committee (LREC) approved the study, which was performed according to the Declaration of Helsinki.

**Table 2:** Demographic and clinical characteristics of participant groups.

	Patientsn = 30Mean(SD)	Controlsn = 30Mean(SD)	t or χ2 value	p
**Age**	66.4(±10.5)	62.4(±7.5)	−1.7	ns
**Gender(M∶F)**	14∶16	14∶16	0	ns
**Education**	12.8(±1.7)	13.7(±2.4)	1.7	ns
**ACE-R**	88.2(±1.9)	94.7(±4.5)	3.2	<0.01
**MMSE**	28.3(±1.8)	29.4(±1.4)	2.6	ns
**Fluency**	10.5(±2.7)	13(±1.1)	4.5	<0.01
**BDI**	9.2(±5.7)	2.5(±3.8)	−5.3	<0.01
**H&Y**	2.2(±0.6)	-	-	-
**UPDRS total**	34.1(±17.8)	-	-	-
**UPDRS I**	3.1(±2.4)	-	-	-
**UPDRS II**	10.1(±7.7)	-	-	-
**UPDRS III**	23.3(±11.1)	-	-	-

**Education**: education duration in years. **ACE-R**: Addenbrooke's cognitive examination revised. **MMSE**: Mini-Mental State Examination score. **BDI**: Beck depression inventory II. **H&Y**: Hoehn & Yahr stage. **UPDRS**: Unified Parkinson's disease rating scale. p-values refer to chi-squared tests or unpaired student t-tests as appropriate (uncorrected).

Participants underwent a clinical and cognitive assessment including: confirmation of diagnosis (UK brain bank criteria); staging [H&Y, Hoehn & Yahr stage [47]]; UPDRS, Unified Parkinson's disease rating scale [48]]; screening for normal or corrected to normal vision (Snellen eye chart <6/9); MMSE (score: 0–30); Revised Addenbrooke's Cognitive Examination (ACE-R score: 0–100, with 5 subscales: orientation, memory, verbal fluency, language and visuo-spatial abilities [49]); and Beck Depression Inventory (BDI-II score: 0–63).

Participants then completed three types of assessment of impulsivity (see table 3). These included (1) self-report questionnaires covering impulse control disorders, temporal discounting and personality ratings related to impulsivity; (2) neuropsychological measures of impulsivity and inhibition, with verbal and non-verbal material, timed and untimed tests; and (3) a test of saccadic inhibition, using infrared oculography during saccadic NoGo inhibition.

**Table 3:** List of tests used for assessment of impulsivity, including brief description of the task, the available outcome measures, and the measures included in the principal components analysis.

Test Type	Test and reference	Brief description	Outcome measures or subscales	Principal measure included in PCA
1	Kirby Temporal DiscountingKirby and Marakovic [130]	Serial forced choice between two rewards, of varyingMagnitude and different time delays	*k* values	k
1	BIS Barrat Impulsivity ScaleStanford et al. [60],Barrat [131]	30 item self-report questionnaire3 second order factors of the impulsivity concept	1. Attentional Impulsiveness2. Motor Impulsiveness3. Nonplanning Impulsiveness	BIS score
1	Behavioural Inhibitory System Behavioural Approach System BIS/BAS, Carver [132]	Self-Report questionnaire	1._ BAS-Behavioural Approach System for appetitive motivation2._ BIS-Behavioural Avoidance System for aversive motivation	BAS scores
1	South Oaks Gambling Screening –SOCSLesieur and Blume [133]	Structured interview on different aspects of pathological gambling	Total score	na
1	Modified-Minnesota Impulsive Disorders Interview –MIDI, Grant [134]	Structured interview exploring different areas of impulsive, repetitive, explosive and compulsive sexual behaviour	Total score	na
2	Motor Go-NoGo task	“Action restraint” inhibition of a prepotent response in response to a low frequency visual cue	Commission errors on NoGo trialsOmission errors and RT on Go trials	Commission errors
2	Temporal interval estimation	Subjective estimation of short periods of time, producing 2, 5, 9 and 17 s intervals	Temporal scaling error (proportional)	Estimation error
2	Frontal Assessment Battery –FABDubois et al. [135]	Brief clinical assessment of frontal function.Items 4 and 5 asses motor inhibitory control	Total score on items 4–5	Total score
2	Stop-signal Task (CANTAB)	“Action cancellation” Stop signal response task.	Direction of error, percentage successful Stop trials, Go reaction time, Stop signal reaction time (SSRT)	SSRT
2	Cambridge Gambling Task (CANTAB)	Assesses decision-making and risk-taking behaviour within gambling context.	Delay aversion (difference between the risk-taking on descend and ascend conditions), Deliberation time, Quality of decision, Risk taking	Delay aversion
2	Hayling Sentence Completion TestBurgess [136]	Sentence completion	Total score, Scaled score	Total score
2	Stroop test, Stroop [137]	Colour-word conflict resolution (timed)	‘CW’ responses	Total score
3	Saccade NoGoPerneczky et al. [94]	Conditional reflexive horizontal saccades, to either go or no go.	For error rate, mean reciprocal RT, variances, and estimate of express saccades variance	Error rate

Type 1 tests are based on questionnaires or interview and self-reporting, Type 2 tests are behavioural response measures, for manual tasks. Type 3 indicates a saccadometric decision_task.

The manual Go/NoGo task and Time Interval Estimation task were implemented using E-Prime software. In the NoGo task participants completed 3 blocks of 128 trials each of a visuomotor NoGo task. Each trial consisted of a black cross (2000 ms fixation point) followed by a black capital letter (500 ms target) in Courier New 18. Participants were asked to press a response button as fast as possible (Go trials, 82%) except after the letter “X” (NoGo trials, 18%). The interval between presentations of NoGo cues varied in a pseudorandom design, with between 1 and 9 intervening Go trials.

The Stop signal task and the Cambridge Gambling task were from the Cambridge Neuropsychological Test Automated Battery (CANTAB, Cambridge Cognition, UK). For the Stop Signal task, participants pressed a left hand button when they saw a left-pointing arrow and a right hand button when they saw a right-pointing arrow. When they then heard an auditory tone (on 25% trials) they had to try to withhold their response. Over 244 Go trials and 80 stop trials, a staircase function was used to estimate the stop signal reaction time, with 50% stopping success, ensuring that participants were operating at a comparable threshold of their own inhibitory ability [50].

For the Time Interval Estimation task participants had to estimate periods of 2, 5, 9 and 17 seconds, following a 1 second reference tone. The required interval direction was specified by visual presentation 3 seconds after the tone. The subject tapped twice on a button: once to indicate the onset and once to indicate the end of their estimation period. Following the first tap there was a 500 ms refractory period to avoid errors in measuring due to tremor. After 4 practice trials, experimental trials were presented in blocks of 20 trials. At the start of each block, participants were instructed not to count out loud or use any other counting strategies to measure the passing of time (cf. [51]–[53]). Pathological distortions in timing and time perception in the seconds-to-minute range are related to impulsivity [54]. Time estimation errors above 1 s contribute to premature responses, representing a measure of impulsiveness [31], [33].

Saccadometry used direct infra-red oculography (Ober consulting, Poland). For each trial, a head mounted laser projected two central dots (one green, one red) for a randomized duration (1.5–2.5 sec). One of these disappeared, and a new red target appeared at +/−10 degrees horizontal displacement. If the central green dot remained, the subject was required to make a saccade to the lateral target (Go trials, 50%). If the central red dot remained, the subject was required to maintain fixation centrally (NoGO trials, 50%). After the saccade was made with a 250 ms gap, the lateral target disappeared, and the subject returned to central fixation on the adjacent pair of red and green dots. 300 trials were completed, randomising laterality and trial type. Automatic pre-processing of errors and latencies was carried out with subsequent fitting to estimate mean and variance of bimodal recinormal latency distribution using SPIC 2.iii software (www.cudos.ac.uk/spic).

### Statistical Analysis

Statistical analysis was carried out using SPSS for Windows v19.0 (IBM). Demographic data were compared between groups with unpaired t-tests or chi-squared tests where relevant. For each of the eleven impulsivity tests listed in table 3, the principal outcome measure was compared between groups using unpaired two-tailed t-tests.

The main analysis had three aims, (a) to determine significant impulsivity factors by the analysis of variance-covariance for impulsivity tests; (b) to determine the tests that contribute most to the impulsivity factors; and (c) to use factor loadings across subjects to estimate the relationship of these factors to demographic and clinical characteristics.

In order to identify the components of impulsivity in Parkinson's disease we used factor analysis. Dimensional reduction by factor analysis assumes that the observed covariation among measures is due to one or more causal latent variables. These underlying factors (or latent variables) were identified by the principal component method. We included 11 variables, with one main outcome measure from each of the impulsivity tests (table 3). The selection of these independently observed variables was based on the single most relevant metric for some tests, such as the Stop Signal Reaction Time (SSRT) from Stop Signal tasks and commission errors from NoGo tasks. For other tests, a summary score was used, based on a high correlation between the well-defined impulsivity subscales (e.g. the BIS sum of all three high correlated subscales). For yet other tests, the selection was based on its likely relevance to parkinsonian impulsivity according to previous studies: within the Cambridge Gambling task the “Delay aversion” value is a measure of motor impulsivity associated with serotoninergic and dopaminergic systems [31]. For Time estimation tasks, mean variability per estimation range has been related to impulsivity in Parkinson's disease patients [33] and BAS subscales have been previously used to evaluate impulsivity in diseases as Attention deficit hyperactivity disorder [55]. The MIDI scores and South Oaks Gambling scores were omitted because of the very low rates of positive responses across this sample, which prevented these tests from contributing meaningful variance to the analysis.

In the main analysis all sixty subjects were included; both controls and patients would contribute to different factors of impulsivity variance, under the hypothesis that impulsivity variance over the whole group would be dominated by the presence and variable severity of Parkinson's disease. A secondary factor analysis was performed, with Parkinson's disease patients alone.

A scree plot was used to identify the number of significant factors, using Catell's criterion (retaining factors ‘left of the elbow’). All retained factors had their eigenvariate greater than one (Kaiser criterion). For interpretation of factor loadings, a threshold of loading on each test was set at 0.50. To best differentiate the original variables for each extracted factor we used orthogonal rotation (varimax) of factors.

Next, to test the assumption that the whole group analysis was driven primarily by the presence and heterogeneity of Parkinson's disease, we compared the factor loadings across both the whole study group and the Parkinson's disease-only group. The whole-group factors were correlated with the Parkinson's disease-only group factors across patients (using Pearson correlation). Although the order of factors was not identical, each factor in the whole group had a corresponding factor in the Parkinson's disease-only subgroup, with which it correlated highly (see Results).

Finally, we examined whether impulsivity factor loadings across subjects correlated with demographic and disease variables. We investigated the possible contribution of each demographic variable into the total variance using linear regression analyses. Each of the four factors was used as a dependent variable with step-wise linear regression (step-out) beginning with a full explanatory model including age, sex, UPDRS total, UPDRS III motor subscale, MMSE, FAB, ACE total, verbal fluency, years of education and L-dopa equivalent dose. This step-wise procedure accommodates the nesting of potential explanatory variables (MMSE within ACEr, UPDRS-III within UPDRS-total).

## Results

The demographic details and summary of disease status assessments are set out in table 2. The age, education and gender did not differ significantly between groups, although the fluency and BDI differences are typical of Parkinson's disease even in the absence of depression [56].

For the eleven impulsivity tests included in the factor analysis, table 4 summaries the individual test differences between patients and controls. Nine of eleven tests differed between groups, in keeping with the published literature. For saccades, patients made more commission errors and the distributions of correct reflexive saccades were also different: with higher variance (Sigma σ: F_1,53_ = 5.735, *p*<0.02) and a higher rate of correct but premature express saccades (Early-sigma σE, F_1,53_ = 5.122, *p*<0.028).

**Table 4:** Impulsivity variables in patients and controls, showing multiple univariate comparisons (Student t-tests, uncorrected for multiple comparisons) for the scalar variables derived from the tests in table 2.

Test	Variable	PD(mean±SD)	Control(mean±SD)	PD *vs* Cont value	PD *vs* Con*p*<
**Frontal Assessment Battery**	Score (tests 4–5)	5±1.3	5.76±.5	2.9	.005
**Stroop test**	Words completed	78.8±25.1	103.1±20.6	4.0	.001
**Saccade NoGo**	Error	19.9±17.5	12.3±11.9	−4.9	.001
**Barratt Impulsivity Scale (BIS)**	BIS total	64.07±9.8	59.4±8.6	2.5	.014
**Manual Go/NoGo**	Errors	22.14±24.6	10.56±11.8	−2.3	.024
**Stop Signal Task (SST)**	SSRT	251. ±111.0	205.0±41.1	−2.1	.038
**Behavioural Approach System**	BAS	9.4±2.3	8.1±2.1	2.1	.042
**Cambridge Gambling Task**	Delay Aversion	0.27±.3	0.22±.2	−.7	.483
**Time estimation test**	Error	98.2±24.8	83.8±1.3	2.2	.027
**Kirby temporal discounting**	k	0.01±.7	0.14±.8	.7	.486
**Hayling Sentence Completion Test**	scaled score	3.93±1.7	5.73±.8	4.9	.001

The SOGS and MIDI scores were excluded due to the scarcity of positive responses.

The whole-group factor structure was driven predominantly by the presence and variable severity of Parkinson's disease. For the whole group factor analysis, Bartlett's test of sphericity (χ^2^(55) = 83, *p*<0.01) indicated that the covariance matrix was not close to identity. There were four significant components which together explained 60% of total variance. Table 5 shows the factor loadings following varimax rotation.

**Table 5:** Component matrices from the whole-group principal components analysis (controls and Parkinson's disease patients), showing the percentage of variance explained by each orthogonal impulsivity factor and their eigenvalues.

Component Test	Impulsivity 1	Impulsivity 2	Impulsivity 3	Impulsivity 4
**Frontal Assessment Battery**	**.850**	0.57	−.030	−.098
**Stroop**	**.708**	.191	.034	.352
**Saccade NoGo error %**	**−.755**	.225	.183	−.074
**BIS score**	**−.573**	−.055	−.046	−.301
**Manual NoGo error %**	−.013	**.804**	.062	.112
**SST-RT**	−.174	**.735**	.034	−.017
**BAS score**	.291	**.583**	−.354	−.140
**Delay aversion**	.026	−.046	**.873**	.077
**Time Estimation %**	.155	−.054	**−.599**	.352
**Temporal discounting k**	−.088	.221	−.099	**.789**
**Hayling score**	.295	−.246	−.002	**.634**
**Variance explained**	18.6%	17%	13%	13%
**Eigenvalue**	2.1	1.7	1.2	1.1

Loadings of each task on the four factors are shown after varimax rotation, and shown in bold above the threshold 0.5.

Regression analysis verified that the first factor of impulsivity was associated with more severe disease (UPDRS-III) and longer disease duration as well as less education and lower cognitive function. The second factor of impulsivity was associated with lower cognitive function, higher levodopa dose equivalent and more severe motor parkinsonian symptoms (UPDRS-III). The third factor was associated with more severe motor signs, longer duration and higher levodopa dose equivalent. The fourth factor was associated with male gender.

Parametric correlations of factor loadings across subjects for the two factor analyses revealed that each of the whole-group factors correlated with one of the factors identified from the Parkinson's disease-only group. Specifically, the whole-group first factor was correlated to the Parkinson's disease group second factor *r* = .885, *p*<0.0001. The second whole-group factor correlated to the Parkinson's disease group first factor *r* = .929, *p*<0.0001. The third whole-group factor correlated with the Parkinson's disease group third factor *r* = .870, *p*<0.0001. The whole-group fourth factor correlated with the Parkinson's disease group fifth factor *r* = .656, *p*<0.05. This suggests that the whole-group factor structure is driven predominantly by the presence and variable severity of Parkinson's disease.

## Discussion

This study has shown that impulsivity in Parkinson's disease is not a unitary phenomenon but multifactorial. We confirmed previous reports that many individual measures of impulsivity are impaired in Parkinson's disease [25], [30], [57]–[59], across a wide range of test types. However, a factor analysis revealed four orthogonal factors, each loading on to a subset of individual tests. Moreover, these four factors had distinct relationships to demographic and disease variables. Subsidiary analyses indicated that the factor structure was driven by the presence and heterogeneity of Parkinson's disease.

Impulsivity is known to be a complex construct [60], a fact which is reflected in its extended definitions [1], [61], [62]. Among the four factors we identified, the percentages of variance explained were similar (range 13–18%). Although there was evidence for a “generalized inhibitory deficit in PD” [27] our data suggest that there is not a unitary mechanism or even a continuous behavioural spectrum of impulsivity in Parkinson's disease [57], [63]. Within each of the factors there were tests that differed between controls and patients in univariate comparisons. Importantly, these differences were observed even in a population that did not include patients with current impulse control disorders.

In the introduction, we suggested several independent potential mechanisms of impulse control deficits, arising from dopaminergic, serotonergic, noradrenergic neurotransmitter dysfunctions and regional structural changes. Although this study does not selectivity manipulate these systems or measure receptor density or brain structure, our proposal (illustrated in figure 1) is largely supported by the factor analysis, in combination with previous psychopharmacological studies (see table 1). We have identified four impulsivity factors, which we will discuss in turn.

The first impulsivity factor (see Table 5) was weighted towards tests of interference in decision tasks, namey the commission errors when deciding whether to make saccades; the subscales of the Frontal Assessment Battery; and the Stroop colour-word performance. These tasks are linked by their demands on conflict resolution, especially tasks where interference is present on a high proportion of trials (50%). The inhibition associated with this first factor is therefore associated with the high frequency of events that require a change of a response. This contrasts with the low frequency events in the manual NoGo task and SSRT (the second impulsivity factor, below). From the correlations with factor loadings, we found that the first impulsivity factor was associated with more severe motor symptoms, longer disease duration, lower cognitive performance and fewer educational years, but not levodopa dose equivalent (see Table 6). The first factor was also associated with the self-assessment of impulsive behaviours, on the Barrett Impulsivity Scale, suggesting that the neurobiological substrate for this factor is manifest in widely differing test modalities. Two potential contributors to this first factor in Parkinson's disease are structural integrity of the prefrontal cortex [42], [64] and change in underlying frontal white matter [65]. These correspond to regions in which damage or atrophy [66]–[69] have been linked to poor Stroop task performance, impaired saccade inhibition and impulsivity.

**Table 6:** Stepwise linear regression for the four impulsivity factors and clinical or demographic variables.

Impulsivity factors	Variables	Standardized coefficients/Beta	t value	*p*<
**F1**	UPDRS_III	−.360	−3.522	.001
	Education	.305	3.445	.001
	Duration	−.276	−3.136	.003
	ACE Total	.254	2.520	.014
**F2**	ACE Total	−.256	−2.171	.033
	l-dopa (LED)	−.254	−2.562	.013
	UPDRS_III	.278	2.374	.020
**F3**	UPDRS_III	.330	3.080	.003
	Duration	−.280	−2.601	.011
	l-dopa (LED)	.238	2.220	.030
**F4**	Gender (M)	.305	2.826	.006

For each row, the impulsivity factor corresponds to the four factors in table 5. UPDRS: Unified Parkinson's disease rating scale. ACE-R: Addenbrooke's cognitive examination revised. LED: levodopa dose equivalent.

The second factor (see Table 5) included tests of motor response inhibition, both action restraint and action cancellation. Both Stop-signal and NoGo tasks are associated with the activity or integrity of the right inferior frontal function [13], [50], [70]. Changes in white matter beneath this region in Parkinson's disease [65] may underlie this factor. However, in the introduction, we drew attention to the fact that these two tests can be differentiated from selective neurochemical dissociations in studies of animals and healthy humans, contrasting serotonergic and noradrenergic functions [1], [12], [14], [71]. In our cohort, both types of inhibition may have been factored together because of the shared role of dopamine. Neurocomputational and neurochemical studies reveal that intra-striatal dopamine mechanisms contribute to both NoGo performance and to the Stop signal task [72], [73] although the dorsal striatal D1 and D2 receptors mediate opposite effects on inhibition [74], and systemic levodopa does not always modulate these forms of inhibition [75]. Such a role for striatal dopaminergic modulation of motor inhibition is consistent with the observed association between our second impulsivity factor and the levodopa daily dose equivalent.

It could be argued that the correlation of this second factor loading with levodopa dose equivalent is an epiphenomenon related to a common association of levodopa dose with disease severity. However, this explanation is unlikely as greater motor impairment severity and higher levodopa dose equivalent had opposite effects on this second factor. It is also possible that the dopaminergic effect is manifest indirectly, through dopaminergic interactions with serotonin [76], [77]. Selective intervention studies with PET imaging may help to determine the specific dopaminergic contribution to this mode of impulsivity.

The second factor includes both objective behavioural tests of motor impulsivity and a self-report questionnaire (BAS). If these two forms of test are mechanistically linked, then the BAS score may be more appropriate in large scale population studies of this mode of impulsivity, while the objective SSRT and NoGo motor tasks enable a translational bridge to comparative studies of impulsivity and model systems for novel treatments.

The third factor was weighted towards Delay Aversion on the Cambridge Gambling Task and time estimation bias. This is consistent with earlier studies of time estimation [54], [78] and delay aversion in Parkinson's disease [31]. The gambling errors may be driven by temporal distortion [79]–[81], since Parkinson's disease increases premature responses without impairing the rational decision making between bets of different value. Although our cohort did not have impulse control disorders, impulsivity on the gambling task is exacerbated by impulse control disorders [82] and dopaminergic therapy [31], [83]. It is relevant therefore that this third impulsivity factor was associated with the participants' levodopa dose equivalent. Interestingly, the promotion of dopaminergic function by amphetamine distorts temporal estimation [84], [85]. How can this effect be reconciled with the positive correlation with disease severity in the current study? We suggest that the relative mesocortical hyperdopaminergic state in early Parkinson's disease [86] leads to dopamine-overdose when in the ON state [31], [87]–[89].

The fourth and final impulsivity factor we identified was weighted towards temporal discounting, the tendency to undervalue future rewards and overvalue immediate rewards. This is not a result of errors in short interval estimation, but a preference for the *relative* immediacy of outcomes. A preference for earlier outcomes has been linked with impulse control disorders and Parkinson's disease [82]. This factor was associated with male gender, consistent with the epidemiological data showing that men are at greater risk of impulse control disorders [3]. This defect in intertemporal choice may be a feature of Parkinson's disease but it was not worsened by dopaminergic medication [90], consistent with the lack of association of the fourth factor with dopaminergic dose equivalent. The non-significant univariate contrast between patients and controls, but significant factors loading, suggests that intertemporal choice changes subtly in Parkinson's disease patients who do not have impulse control disorders.

The fourth factor is also expressed in terms of performance on the Hayling test, in which an immediate prepotent response must be replaced by a less obvious novel answer. This might in principle relate to the verbal fluency deficit in Parkinson's disease [56], [91]. Against a simplistic fluency argument is the need for only a single word response on each question on the Hayling test and the lack of significant fluency deficits in our cohort. The Hayling test could also be considered to invoke interference between correct and prepotent responses, with monitoring and resolution of response conflicts for accurate and fast responses. As such it would seem to be aligned with the tests in the first impulsivity factor. However, both intertemporal choice and Hayling tests in factor 4 require a response to a hypothetical scenario. These scenarios require the ability to introspect. A failure to do so has been linked to Reflection Impulsivity [92] and adult psychopathology, although it has not been studied before in Parkinson's disease.

Although each of the tests included here has previously been studied in the context of Parkinson's disease, we have demonstrated the relationships between these impulsivity tests. There are two key advantages in determining these relationships.

First, our analysis provides clear evidence of the fractionation of impulsivity in the context of Parkinson's disease. By understanding common factors onto which multiple tests can be mapped, one is able to select representative tests for examining each aspect of impulsivity. Whether such a subset of tests should be based on self-report questionnaires that can be undertaken remotely by subjects, or by laboratory based objective measures, will depend on the immediate purpose of a study. The optimal set of tests for large sample phenotype-genotype correlations may be different from the best choice for a psychopharmacological study, or the optimal set for translational studies between human and animal models. Nonetheless, a time- and cost-efficient subset of tests can be selected that includes each principal component of impulsivity, for the clinical or research setting.

Second, the examination of the factor structure of impulsivity, its demographic and clinical correlates, may lead to specific hypotheses about the underlying anatomical and psychopharmacological mechanisms of impulsivities in Parkinson's disease. Our results suggest that no single drug acting on one neurotransmitter system can be expected to normalise impulsivity in Parkinson's disease. Instead, there is the prospect of a complex, but better informed approach to individualised or stratified treatment. Such a stratified approach to patients could consider a patient's stage, severity, medication and impulsivity profile on tests drawn from the four impulsivity factors. Knowledge of the impulsivity factor can also assist the development of novel cognitive biomarkers for Parkinson's disease. If one mode of impulsivity is to be investigated repeatedly, over time or across treatment conditions, it would help to select a test related to this mode which is objective and highly reproducible with minimal practice effects. For example, in evaluating a therapy for the non-dopaminergic impulsivity factor one, saccade inhibition might be a better index than the FAB, BIS or Stroop tasks (cf. [93], [94]).

### Limitations and future directions

There are methodological, statistical, and inferential limitations of this study. We identified four orthogonal impulsivity factors but cannot exclude the possibility of additional factors, identifiable from a larger study population or a larger battery of tests. However, the selection of tests for our neuropsychological battery covers a wide range of the assessments previously reported in Parkinson's disease literature. We did not use these tests to categorise our patients into impulsive *vs* non-impulsive cases. This reluctance to impose categorical analysis was not only motivated by concerns about statistical power, but by the hypothesis that impulsivity varies as a continuous variable throughout patient populations. The clinical distinction between impulse-control disorder versus non-impulse-control disorder cases may be useful to measure the frequency and causes of impulse control disorders, but it is an artificial and potentially non-biological dichotomy. In this respect, the lack of impulse control disorders in our cohort does not undermine the relevance of our group differences to the broader problem of impulse control in Parkinson's disease. An additional issue in relation to the assessment battery was the potential overlap in some of the selected tests, i.e. MMSE and ACE-R, or UPDRS and its part-III motor subscale. However, these tests were not direct tests of impulsivity, but treated as independent clinical variables which we included only in the final stages of regression of factor loadings onto clinical and demographic variables.

Future studies could include larger samples. PCA benefits from large samples and requires a number of observations at least 5 times the number of variables analysed to obtain reliable results [95]. Our sample size meets this criterion but the inclusion of further cases would increase the robustness of results and enable the inclusion of separate subscales from the neuropsychological tests. Larger populations would also increase the likelihood of including patients with impulse control disorders, and enable one to investigate contributory factors such as medication subtypes (especially use of dopamine agonists). However, it is remarkable that even in a cohort without clinical impulse control disorders, our results show poor inhibitory control on a wide range of tests and reveal the multidimensionality of impulsivity. All our patients were on dopaminergic medication, the mainstay of Parkinson's disease treatment. This fact could affect the estimation of dopamine dependency on impulsivity measures. To offset this, our sample included a wide range of patients in terms of severity of disease and dose equivalent.

A final concern could be the misevaluation of latent symptoms as depression or sleep deprivation that may be affecting impulsivity outcomes. Certainly, the BDI score was higher in patients but the regression analysis did not show any correlation of the BDI with our four impulsivity factors. Our inclusion criterion on the BDI was high (>18), so as to retain relevance to the broader population of Parkinson's disease while excluding severe depression. We did not assess poor sleep quality or fatigue, although these are common symptoms [96] and might relate to impulsivity [97]. Rather, we sought to minimise fatigue during testing, by frequent breaks and refreshment during the sessions.

In summary, the study has confirmed that impulsivity is a complex construct, and that Parkinson's disease affects several different modes of impulsivity. We have shown how four factors are differentially related to common clinical, demographic and therapeutic variables. The results reveal impulsivity even in the absence of impulse control disorders, but we emphasise that pervasive and generalised deficits in inhibitory control are not evidence of a unitary deficit. Future therapeutic and biomarker studies of impulsivity in Parkinson's disease can exploit the factor structure of impulsivity, aiding an informed choice of tests pertaining to each factor, whether using subjective questionnaires or objective behavioural tests. We hope that our results will accelerate the comprehensive understanding of behavioural change in Parkinson's disease and progress to more effective and individualised treatments.
